# *Clonostachys rosea*, a Pathogen of Brown Rot in *Gastrodia elata* in China

**DOI:** 10.3390/biology13090730

**Published:** 2024-09-17

**Authors:** Huan Yao, Kang Liu, Lei Peng, Touli Huang, Jinzhen Shi, Beilin Sun, Juan Zou

**Affiliations:** 1 Key Laboratory of Research and Utilization of Ethnomedicinal Plant Resources of Hunan Province, Huaihua University, Huaihua 418000, China; 2 Hunan Provincial Higher Education Key Laboratory of Intensive Processing Research on Mountain Ecological Food, College of Biological and Food Engineering, Huaihua University, Huaihua 418000, China; 3 College of Biological and Food Engineering, Huaihua University, Huaihua 418000, China; yh15574539680@163.com (H.Y.); lk2195138947@163.com (K.L.); 15581590618@163.com (L.P.); htlsznbd20050207@126.com (T.H.); 18526725911@163.com (J.S.); sbl2200120525@outlook.com (B.S.)

**Keywords:** *Gastrodia elata*, brown rot, *Clonostachys rosea*, morphology, phylogenetic analysis

## Abstract

**Simple Summary:**

The tubers of *Gastrodia elata*, used in traditional medicine for over two thousand years, are highly susceptible to fungal infections, making tuber rot a common issue during its cultivation. This study investigated a significant outbreak of brown rot disease affecting *G. elata* f. *elata* in Hunan Province, China. Typical symptoms include initial brown spots on the tuber surface that expand and darken, with the spots spreading inward and causing the surrounding tissue to transition from brown to black. In this study, the fungal pathogen causing brown rot disease on *G. elata* was isolated and examined in detail for morphological characteristics, including color, sporulation structures, and spore size. Its identification was further supported by phylogenetic analysis. The findings indicated that the fungal pathogen causing brown rot disease on *G. elata* f. *elata* was *Clonostachys rosea*.

**Abstract:**

*Gastrodia elata*, commonly known as Tian Ma, is a perennial mycoheterotrophic orchid. Qianyang Tian Ma (QTM), a geographical indication agricultural product from Hongjiang City, Hunan Province, China, is primarily characterized by the red variety, *G. elata* f. *elata*. A severe outbreak of tuber brown rot disease was documented in QTM during the harvesting season in Hunan. The fungal pathogen associated with the disease was isolated on potato saccharose agar (PSA) and identified through morphological and phylogenetic analyses. Pathogenicity tests were performed on healthy tubers of *G. elata* f. *elata*. The results showed that the representative isolate, named TMB, produced white hyphal colonies with a ring structure, broom-like phialides, partially curved ellipsoidal conidia, and orange–yellow spherical ascocarps on PSA. Phylogenetic analysis of the ITS, LSU, *rpb2* and *tub2* sequences using Bayesian and maximum-likelihood methods identified the isolate TMB as *Clonostachys rosea*, based on morphological and phylogenetic data. Pathogenicity tests revealed typical disease symptoms on healthy *G. elata* tubers 15 days post-inoculation with the isolate TMB. *C. rosea* is known to cause diseases in economically important crops, but there are no reports of its occurrence on *G. elata* f. *elata* in China. This study provides valuable insights into the occurrence, prevention, and control of brown rot disease in *G. elata* f. *elata*.

## 1. Introduction

*Gastrodia elata* Bl., commonly known as Tian Ma, is a perennial herbaceous plant in the Orchidaceae family with highly degenerated roots and leaves, relying on the mycelium of the parasitic fungus *Armillaria mellea* for nutrition after seed germination due to its inability to absorb soil nutrients or perform photosynthesis [1,2]. It is distributed from the Russian Far East through China, Japan, Korea, Southeast Asia, and countries south of the Himalayas, extending to India, New Guinea, Australia, New Caledonia, Sri Lanka, and Madagascar. In China, *G*. *elata* is known by various names such as Chi Jian, Du Yao Zhi, He Li Cao, and Bai Long Pi and is recognized as a traditional medicinal plant [3]. It is widely distributed across the Yunnan, Sichuan, Guizhou, Shaanxi, and Jilin provinces, as well as the eastern and northwestern regions of Liaoning, predominantly growing at altitudes ranging from 400 to 3200 m in sparse forests, forest clearings, forest edges, and shrub fringes [4]. Based on quality and color, *G. elata* can be classified into nine synonyms: *G. elata* f. *elata*, *G. elata* f. *viridis*, *G. elata* f. *flavida*, *G. elata* f. *glauca*, *G. elata* f. *alba*, *G. elata* f. *pilifera*, *G. elata* var. *gracilis*, *G. elata* var. *pallens*, and *G. elata* var. *viridis* [2]. The tuber of *G*. *elata*, which has been used medicinally for over two thousand years, is known for containing various chemical compounds, with the primary active ingredients being identified as gastrodin, polysaccharides, p-hydroxybenzyl alcohol (gastrodigenin), and others [2,3,5]. Gao et al. (2022) reported that the active components of *G*. *elata* alleviated depression in CUMS-induced mice by reducing hippocampal neuronal apoptosis, enhancing 5-HT and DA levels, lowering Ca^2+^ concentration and MAO activity, and modulating the expression of BDNF and NMDAR compared to controls [6]. Gan et al. (2024) found that *G. elata* polysaccharide significantly alleviated motor dysfunction, inhibited α-synuclein accumulation, and reduced dopaminergic neuron loss in mice with Parkinson’s disease by increasing the Bcl-2/Bax ratio, decreasing cleaved-caspase-3 levels, and reducing GFAP, TNF-α, IL-1β, and IL-6 levels through the TLR4/NF-κB pathway [7]. Wu et al. (2024) demonstrated that *G. elata* polysaccharide protected against myocardial injury in sleep-deprived mice by inhibiting ferroptosis via SIRT6, as evidenced by reduced ROS levels, improved oxidative stress markers, and the modulation of key ferroptosis-related proteins, with these effects reversed by the SIRT6 inhibitor OSS_128167 [8]. Fu et al. (2023) demonstrated that Gas-miR2-3p, a specific miRNA derived from *G. elata*, alleviated neuroinflammation by downregulating inflammatory factors such as TNF-α, IL-6, and IL-1β and by inhibiting the activation of the NF-κB signaling pathway [9]. Yu X et al. (2024) revealed that p-hydroxybenzaldehyde (p-HBA), one of the main active components of *G. elata*, enhanced antioxidant effects, delayed aging, and helped in the treatment and prevention of Alzheimer’s disease by reducing reactive oxygen species (ROS), lowering lipofuscin levels, and inhibiting β-amyloid aggregation [10]. Additionally, *G. elata* is highly nutritious, with its tubers being rich in protein, starch, and dietary fiber while being low in fat and also containing high levels of trace elements such as cobalt, iron, manganese, zinc, and selenium, which are essential for maintaining health and supporting metabolism [5,11,12].

The tubers of *G. elata* are susceptible to infection by a variety of fungal species, with tuber rot being a prevalent issue during both cultivation and growth [13]. Li et al. (2022) identified *Fusarium redolens* as the pathogen causing tuber rot in *G. elata* in Guizhou Province, China, with infection rates ranging from 10% to 25% [14]. In 2018, root rot disease in *G. elata* from Gimcheon, Korea, was confirmed to be caused by *F. oxysporum* through morphological characteristics and molecular analysis [15]. Li et al. (2022) reported that *Botrytis cinerea* is responsible for causing gray mold on the flowers of *G. elata* [16]. Tang et al. (2022) used ITS and 16S rDNA gene analysis to identify microorganisms associated with tuber rot in *G. elata*, revealing that the genus *Ilyonectria*, particularly *I. cyclaminicola* and *I. robusta*, is closely related to the disease, with an isolation frequency of 42.0% [17]. These fungi not only cause tuber rot in *G. elata* but also inhibit the growth of the symbiotic fungus *A. mellea*. Overall, *G. elata* tuber diseases can be categorized into two main types: spot rot and wet rot. Spot rot is characterized by surface dehydration, wrinkling, and the formation of small black spots that progressively enlarge, resulting in a blackened appearance with internal necrosis along the central vascular bundles. Wet rot features water-soaked lesions without rust spots, leading to rapid tuber decay and a foul odor.

In Hunan Province, the renowned Qianyang Tian Ma (QTM), primary cultivar *G. elata* f. *elata*, was designated as a geographical indication agricultural product of Hongjiang City in 2020, with its geographical protection scope encompassing a broad area within Hongjiang City, covering 84 administrative villages in 16 townships and towns, including Anjiang Town, Taiping Township, Chatou Township, Maodu Township, Qiancheng Town, Jiangshi Town, Xuefeng Town, Tangwan Town, Tieshan Township, Qunfeng Township, Wanxi Township, Xima Township, Dachong Township, Shuping Township, Longchuantang Yao Township, Shendu Miao Township, as well as Xuefeng Mountain forest farm and Bamian Mountain farm [18]. In addition to local markets in Hunan, the product is also exported to nearby regions such as Guizhou and Guangxi, and even to Southeast Asia [19]. In recent years, the expansion and prolongation of *G. elata* cultivation have intensified problems related to continuous cropping systems. Among these issues, brown rot has emerged as a particularly severe disease. In its early stages, brown rot manifests as near-circular dark-brown lesions on QTM, which progressively enlarge, leading to significant reductions in both yield and quality.

This research investigates the pathogen responsible for brown rot disease in QTM, combining morphological observations with sequence analysis of genes such as ITS, LSU, *rpb2*, and *tub2* to identify the pathogen species. Koch’s postulates are then applied to confirm the pathogenicity of the isolated fungus and verify it as the cause of brown rot in *G. elata*. This study provides a theoretical foundation for biological control methods and supports the development of QTM.

## 2. Materials and Methods

### 2.1. Materials

Diseased tubers of QTM were collected from the Xuefeng Mountains, Shuitian County of Hunan Province in southern China, located at an elevation of 700 m, 27.60° N, 110.20° E.

### 2.2. Fungal Isolation and Cultivation

In total, 12 diseased specimens were gathered from three different locations for laboratory testing. Tissues containing the infected sections of the specimens were sterilized using 75% ethanol for 1 min, followed by immersion in 2.5% hypochlorite for 45 s. After sterilization, the symptomatic samples were rinsed three times with sterile water, placed on potato saccharose agar plates, and incubated at 25 °C for 48 h [20]. The representative isolate, named TMB, was selected for study. The morphological characteristics of the colonies were documented, followed by the examination of pathogen morphology, color, sporulation structure, and measurement of spore size under a light microscope.

All the experiments were conducted in triplicate by default, with the spore size measured for 50 samples. Statistical significance was determined using SPSS 17.0 (SPSS Inc., Chicago, IL, USA). The results are presented as mean ± standard deviation (SD).

### 2.3. Molecular Phylogenetic Analyses

A 1 cm × 1 cm clot was inoculated into 50 mL of PSA liquid medium in a 250 mL flask and incubated at 25 °C and 150 r/min for 48 h. The hyphae were collected by centrifugation at 9800× *g* for 5 min, and the DNA extraction protocol was as follows: 200 mg of fresh mycelium was powdered using a mortar and pestle in liquid nitrogen, mixed with 500 µL of SDS extraction buffer (containing 100 mM Tris-HCl, 500 mM NaCl and 50 mM EDTA, pH8.5), and subsequently incubated at 65 °C for 10 min. Next, 150 µL of 5 mol/L KAc was added, followed by chilling on ice for 20–30 min. Centrifugation at 12,000 r/min for 10 min separated the supernatant, which was then mixed with 0.7 volumes of pre-cooled isopropanol for precipitation at 4 °C for 30 min. Genomic DNA precipitation was recovered by centrifugation. After air-drying, the DNA was dissolved in sterilized ddH_2_O. An equal volume of phenol and chloroform mixture was added, followed by centrifugation at 12,000 r/min for 8 min. The supernatant was treated by adding 2 volumes of pre-cooled ethanol, and precipitation was allowed to occur at 4 °C for 30 min. Genomic DNA was recovered post-centrifugation, and the precipitate was washed twice with 400 µL of 70% ethanol. Finally, the genomic DNA was lysed using 200 µL of sterile ddH_2_O.

The internal transcribed spacer (ITS) region (including the partial ITS1 sequence, the 5.8S, and ITS2 complete sequences), the large subunit (LSU) rRNA gene, the RNA polymerase II second largest subunit gene (*rpb2*), and the beta-tubulin gene (*tub2*) were amplified from strain TMB using the primer pairs ITS5/ITS4, LROR-2/LR7, *rpb2*-7cR/5f2, and Bt2a/Bt2b, respectively [21,22]. PCR amplification was conducted with a 25 μL reaction volume comprising 1 μL of template DNA, 9.5 μL of ddH_2_O, 12.5 μL of 2 × T5 Super PCR Mix, and 1 μL each of forward and reverse primers. 

Subsequently, multiple sequence alignments were carried out using ClustalX 2.1 [20]. The ITS, LSU, *rpb2,* and *tub2* sequences were combined via SequenceMatrix 1.8. The compatibility among the four genes was assessed using PAUP 4.0b10 (Swofford, 2002), indicating that the phylogenetic signals for the four loci were consistent (*p* = 0.05), suggesting they align well in the phylogenetic tree. The best-fit model and parameters were calculated with MrModelTest 2.3, and a phylogenetic analysis was conducted employing the Bayesian (MrBayes-3.2.7a) and maximum-likelihood (RAxML 1.0) methods [23]. Branches with bootstrap support values of at least 50% for maximum likelihood (ML) and Bayesian posterior probabilities (BPPs) of 0.70 or higher were considered significantly supported [24,25].

### 2.4. Pathogenicity Testing

Pathogenicity assessments were performed on healthy QTM tubers to fulfill Koch’s postulates. Three tubers were initially surface-sterilized using 75% ethanol and then inoculated by spraying the entire tubers until run-off with a spore suspension of isolate TMB (1 × 10^6^ conidia/mL), while three other tubers served as controls, sprayed with sterile water. After soaking, all the tubers were placed in sterile soil, kept in darkness, and incubated for 15 days at 25 °C.

## 3. Results

### 3.1. Investigation of Brown Rot Disease in QTM

**Figure 1:**
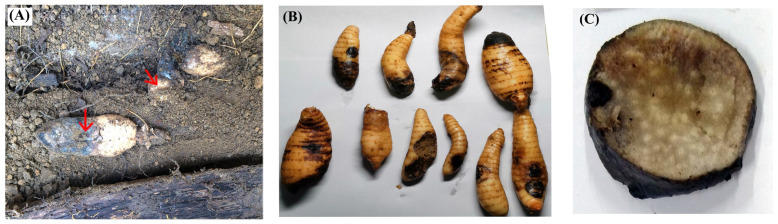

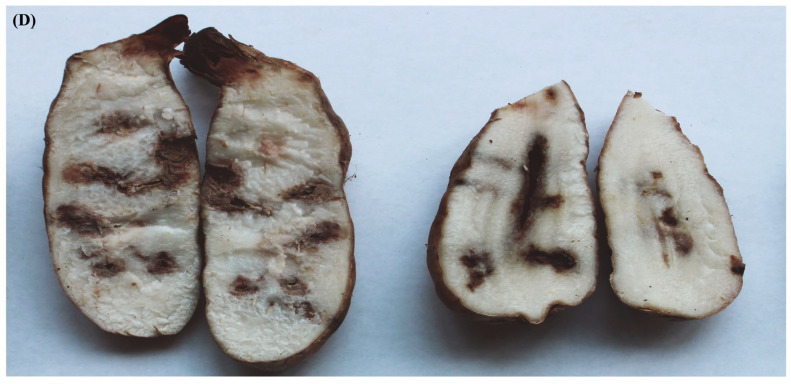
Brown rot disease in QTM. (**A**) Pathogen-contaminated fungal material spreading disease to QTM (as indicated by the red arrows); (**B**) infected QTM; (**C**,**D**) internal anatomical sections of diseased QTM.

Qianyang Tian Ma (QTM), a geographically indicated agricultural product from Hongjiang City in Huaihua, Hunan Province, China, is renowned for its substantial economic and medicinal value. The survey on Qianyang Tian Ma from 2020 to 2023 showed that the crop failure rate ranged from 10% to 25%, while the rot rate ranged from 15% to 55%. Healthy QTM tubers have intact yellow outer skins, whereas infected ones display prominent brown spots on the outer skin in the infected areas, often accompanied by varying degrees of cavities or lesions. The pathogen infiltrates inward through the thin-walled tissues, causing the surrounding tissue to transition from brown to black, ultimately resulting in the entire tuber becoming blackened (Figure 1).

### 3.2. Morphological Characteristics of the Pathogen

**Figure 2:**
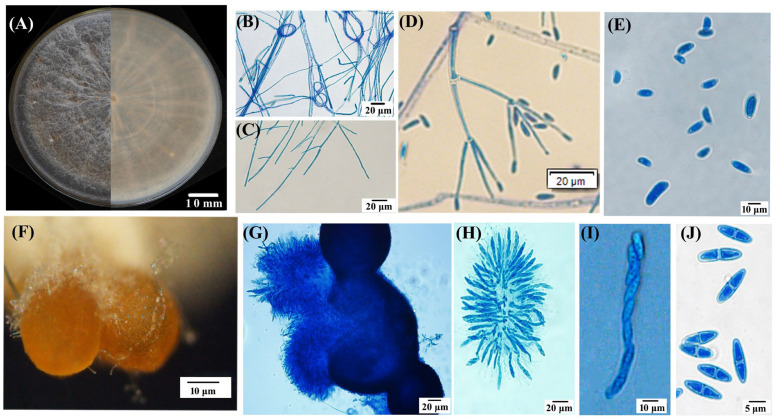
Morphological characteristics of the representative isolate TMB. (**A**) Colonies cultured on PSA medium for 25 days, showing both front and reverse views; (**B**) hyphal ring structure; (**C**) hyphal morphology; (**D**) primary (Verticillium-like) conidiophores and conidia; (**E**) conidial morphology; (**F**) aggregated perithecia grown on a PSA plate; (**G**) perithecium structure; (**H**) asci; (**I**,**J**) ascospores. Scale bar: (**A**) = 10 mm; (**B**–**D**,**G**,**H**) = 20 μm; (**E**,**F**,**I**) = 10 μm; (**J**) = 5 μm.

Nine out of the twelve fungal isolates separated from symptomatic lesions exhibited a similar morphology. The isolate TMB, as a representative, was used for this study. The isolate TMB cultured on a PSA medium for 11 days exhibited slow growth with concentric rings, smooth edges, a predominantly white color with a slight yellow pigment on the reverse side, sparse woolly mycelium, and a measured colony diameter of 6.1 ± 0.3 cm. After 21 days, the aerial mycelium decreased, and brown, granular ascocarp clusters began to appear (Figure 2A). The TMB mycelium possessed septate hyphae and a ring-like structure (Figure 2B,C). The PSA agar blocks covered with mycelium were submerged in sterile water for hydroponic culture, and after one week of incubation at 28 °C in darkness, secondary conidia developed on conidiophores that branched like brooms, exhibiting slightly swollen tips and small protrusions; the conidia were elliptical, slightly curved, and varied in size, 7.8–13.0 × 3.9–7.8 μm (x = (11.2 ± 2.2) × (5.4 ± 1.1) μm, n = 50) (Figure 2D,E). The ascocarps of TMB were approximately spherical, yellow–brown to brown in color, with a diameter ranging from 25.1 to 40.0 μm, often clustered together and attached to white mycelium at the base (Figure 2F). Upon maturation, the ascocarps increase in size, rupture, and release ascospore masses. Each ascus typically contains eight smooth, septate, spindle-shaped ascospores (6.8–10.7 × 2.4–3.9 μm (x = (9.41 ± 1.2) × (3.56 ± 3.12) μm, n = 50), as illustrated in Figure 2G–J. 

### 3.3. Phylogenetic Analyses

**Figure 3:**
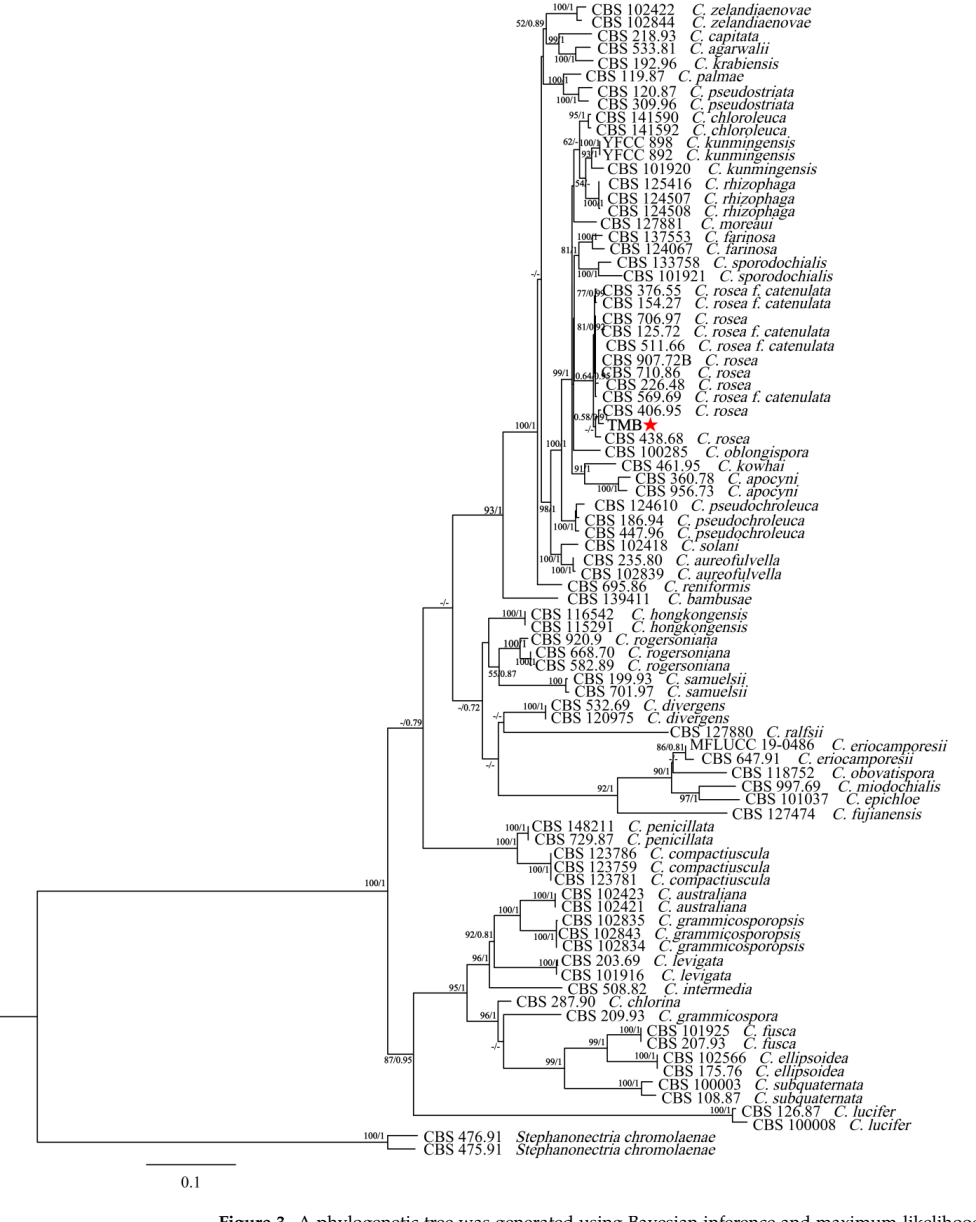
A phylogenetic tree was generated using Bayesian inference and maximum-likelihood analyses of the combined ITS + LSU + *rpb2* + *tub2* dataset. Branch support values (ML/BI) are indicated for nodes with more than 50% bootstrap support and Bayesian posterior probabilities (BPPs) exceeding 0.70. Isolates in this study are highlighted in bold type and marked with a red asterisk.

The new sequence data for the TMB isolate have been deposited in GenBank under the following accession numbers: ITS (PP994883), LSU (PP994885), *rpb2* (PQ009939), and *tub* (PQ009940). The species, specimens, and GenBank accession numbers of sequences used in this study are detailed in Table 1. The ITS + nLSU + *rpb2* + *tub2* dataset included sequences from 86 fungal specimens representing 43 species. The dataset had an aligned length of 2535 characters that were parsimony informative. The maximum-likelihood (ML) analysis tree was generated using RAxML1.3.1 with the GTR+I+G model parameters determined by MrModelTest, based on 1000 bootstrap replicates. The Bayesian inference (BI) analysis was conducted using MrBayes v3.2.7a for 2 million generations, yielding an average standard deviation of split frequencies of 0.006685. The result indicated good convergence of the analysis, suggesting that the posterior probability distribution is reliable and the phylogenetic tree generated is robust. Figure 3 illustrates that the dataset, comprising 42 species of the genus *Clonostachys*, included the isolate TMB, which, along with *C. rosea*, formed a separate clade from the other species with strong support (100% ML bootstrap and 1.00 BPP). The genetic distance between the isolate TMB and *C. rosea* was the shortest among other *Clonostachys* species. Additionally, TMB had the smallest genetic distance with CBS 406.95 *C. rosea*, and the node at which the isolate TMB and CBS 406.95 C. rosea belonged to the same branch had a maximum-likelihood support rate (ML/BI) over 50%, with a Bayesian posterior probability of 0.91. Morphological and phylogenetic analyses revealed that the isolate TMB, distinguished by its unique characteristics and phylogenetic profile, was classified as *Clonostachys rosea*.

### 3.4. Pathogenicity Results

**Figure 4:**
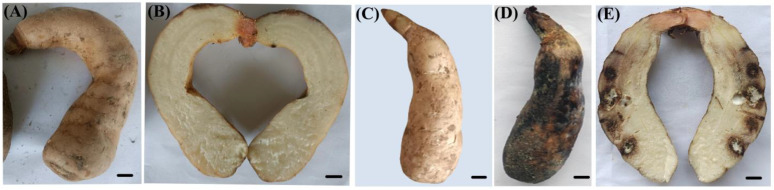
Pathogenicity test of the isolate TMB on *G. elata* tubers. (**A**,**B**) The control tuber was inoculated with sterile water; (**C**) healthy tuber before inoculation with conidia of *C. rosea*; (**D**,**E**) healthy tuber inoculated with conidia of *C. rosea* after 15 days. Scale bar = 1 cm.

After 15 days of cultivation, the *G. elata* tubers were removed from the culture bottles. As depicted in Figure 4, the infected tubers lost their normal fresh color, shifting from light yellow to dark brown. Compared to the control group, the affected areas were notably dull, showing yellow–brown or dark-brown discoloration. Mucilage secreted on the surface of the tubers adhered to the surrounding soil particles. After cutting open the tubers, as shown in Figure 4, brown patches with a central white mycelium were visible inside. The natural disease symptoms were consistent with those induced under laboratory conditions through inoculation. In the control group, no disease symptoms were observed. Isolates reisolated from the inoculated tubers displayed morphological characteristics identical to the original isolate TMB and were confirmed to be molecularly identical. Therefore, it can be concluded that TMB (*Clonostachys rosea*) causes rot in *G. elata* tubers.

## 4. Discussion

The cultivation area for Qianyang Tian Ma (QTM) is located in the Xuefeng Mountain region of Hunan. The Xuefeng Mountain region in Hunan, situated within the Xuefeng Mountain Range (25°95′ N–29°40′ N, 109°41′ E–112°68′ E), represents the largest mountain range in Hunan and constitutes a major geographic boundary between the second and third topographic steps in China, extending from the Hunan–Guangxi border in the south to Yiyang in the north and oriented in a northeast–southwest direction [26]. The main peak of the Xuefeng Mountain Range, Subao Peak, reaches an elevation of 1934 m and has an annual average temperature of 10–19 °C. July temperatures average approximately 18 °C, providing optimal environmental conditions for the cultivation of *G. elata*. Huaihua City has a long history of *G. elata* cultivation, with extensive planting in counties such as Tongdao, Jingzhou, Huitong, Hongjiang, Zhongfang, and Xupu along the Xuefeng Mountain Range, where local residents continue to employ traditional planting techniques and practices [27]. 

This study focused on investigating the pathogenic fungus responsible for QTM tuber brown rot disease. The morphological characteristics of the representative isolate TMB were analogous to the features of *Clonostachys rosea* (Link) Schroers, Samuels, Seifert & W. Gams (1999: 373) [28]. Further molecular identification and validation using Koch’s postulates confirmed that the isolated representative TMB identified as *Clonostachys rosea* was responsible for the decaying QTM tubers. *C. rosea* was initially documented as a pathogen causing root rot in *G. elata* in 2020, specifically in Chuncheon City, Korea (latitude 37°52′ N, longitude 127°44′ E) [29], characterized by a temperate monsoon climate with typically lower annual precipitation, generally below 800 mm, and shorter rainy seasons [30,31]. In contrast, the QTM studied in Huaihua City, Hunan (latitude 25°52′22″–29°01′25″ N, longitude 108°47′13″–111°06′30″ E), grows in a subtropical monsoon climate, characterized by mean temperatures above 0 °C in the coldest months, annual precipitation exceeding 800 mm, and longer rainy seasons [32,33]. The annotated genome sequence of Korean *G. elata* (GenBank accession number MN026874) revealed a chloroplast genome 74 bp shorter than that of Chinese *G. elata*, with significant sequence variants including 495 SNPs and 75 InDels, in which insertions occurred in seven protein-coding genes (clpP, matK, rps12, rpl2, rpl16, ycf, and ycf2) and deletions in four genes (clpP, rpl16, rps12, and ycf2) [34]. Additionally, while Lee et al. (2020) reported *C. rosea* as the pathogen responsible for root rot in Korean *G. elata*, their study did not include any experimental images, such as those showing the characteristics of *C. rosea*, the infection process, or the pathogen testing results [29]. In contrast, our study on brown rot in QTM provides a detailed account of the entire experimental procedure and a systematic analysis of the isolate TMB identification, thereby offering a more comprehensive and accurate scientific basis for understanding brown rot in *G. elata*.

The colony morphology, ascocarps, conidia, and ascospores of the isolate TMB are similar to those described for *C. rosea* by Schroers et al. [35]. The colony reverse of the isolate TMB on PDA medium appears pale orange, with perithecia containing asci bundles densely crowded on a well-developed stroma, occasionally solitary and arising directly from the mycelium, forming after 21 d of incubation at 25 °C. Compared to the type strain CBS 710.86, the primary (Verticillium-like) conidiophores of the isolate TMB had more branching, with two to three levels of branching, and were dispersed at slightly less-acute angles. The primary (Verticillium-like) conidia of isolate TMB range from 7.8–13.0 × 3.9–7.8 μm, whereas those of the type strain CBS 710.86 range from 4.2–6.6 × 2.0–3.4 μm, indicating that TMB conidia are larger and more elongated than those of CBS 710.86. The ascospores of isolate TMB measure 6.8–10.7 × 2.4–3.9 μm, while those of CBS 710.86 range from 7.4–14.4 × 2.2–4.8 μm, making TMB ascospores shorter and thicker compared to those of CBS 710.86. Compared to isolate CBS 406.95 (after 7 days of cultivation), the isolate TMB displays fewer conidiophore branches, and secondary conidiophores have not yet been observed. Our ML and Bayesian results indicate that *C. rosea* grouped with *C. farinosa*, *C. sporodochialis,* and *C. oblongispora*, consistent with the phylogenetic tree of the genus *Clonostachys* constructed by Wang et al. [21]. The isolate TMB clustered with *C. rosea*, forming a distinct branch with the shortest genetic distance among *Clonostachys* species. Moreover, TMB had the closest genetic distance to *C. rosea* CBS 406.95, with their shared branch showing a maximum-likelihood support rate (ML/BI) over 50% and a Bayesian posterior probability of 0.91. There were several base pair differences between the isolate TMB and CBS 406.95 in the sequences, including three base pair differences in the ITS sequences, three base pair differences in the LSU sequences, two base pair differences in the *tub2* gene, and four base pair differences in the *rpb2* gene.

Currently, *C. rosea* is extensively used in scientific research and agricultural practices as a biocontrol agent against pests and fungal diseases. However, there are relatively few reports on its pathogenicity in plants, underscoring the need for further research to ensure its safe and effective use. *G. elata*, which primarily grows underground, is exposed to soil-borne pathogens over extended periods, making it an important model for studying these pathogens. The current research reports indicate that *C. rosea* can infect a range of hosts (as shown Table 2), including barley (*Hordeum vulgare* L. var. *nudum* Hook. f.) [36], faba bean (*Vicia faba*) in Tarom County, northwestern Iran [37], sugar beet (*Beta vulgaris*) [38], avocado (*Persea americana*) [39], and others. These findings suggest that *C. rosea* has a broad host range but also emphasize the need for a more thorough understanding of its pathogenic mechanisms. Despite its significant potential as a biocontrol agent, its potential pathogenicity needs further evaluation to prevent any adverse effects on agricultural production. Future research should focus on elucidating the pathogenic mechanisms of *C. rosea*, its ecological adaptability, and its effectiveness across different crops. Such studies are crucial for optimizing its use as a biocontrol agent and ensuring its safe and effective application in agriculture.

## 5. Conclusions

To manage disease risks effectively, *G. elata* cultivation should be conducted in uncultivated lands with minimal indigenous fungal populations, implemented with regular crop rotation and avoidance of consecutive planting with susceptible crops. Before planting, the soil should undergo deep tillage. In our experimental study, we found that *C. rosea* exhibits high spore production and strong reproductive capabilities, which facilitates its potential transmission during the asexual reproduction of *G. elata*. Therefore, it is recommended to select seed tubers that are free from wounds and disease spots to minimize the risk of infection. Additionally, sexual reproduction in *G. elata* carries a lower risk of disease and allows the plant to inherit beneficial traits from the parent. Thus, promoting sexual reproduction in *G. elata* could be an effective strategy for managing brown rot disease.

## Figures and Tables

**Table 1:** Species, specimens, and GenBank accession number of sequences used in this study.

Species Name	Sample No.	Site of Origin	GenBank Accession No.			
			ITS	*tub2*	LSU	*RPB2*
*Clonostachys rosea*	TMB	China	PP994883	PQ009940	PP994885	PQ009939
*C. rosea*	CBS 710.86	The Netherlands	AF358235	AF358161	MH873700	OQ927842
*C. rosea*	CBS:706.97	USA	OQ910770	OQ982797	OQ911129	OQ927838
*C. rosea*	CBS:907.72B	Ukraine	OQ910777	OQ982804	MH872320	OQ927845
*C. rosea*	CBS:226.48	The Netherlands	OQ910746	OQ982773	MH867871	OQ927814
*C. rosea*	CBS:438.68	Czech Republic	OQ910761	AF358163	MH870894	OQ927829
*C. rosea*	CBS:406.95	France	OQ910759	AF358167	OQ911118	OQ927827
*C. rosea f. catenulata*	CBS 376.55	The Netherlands	OQ910802	AF358162	MH869057	OQ927868
*C. rosea f. catenulata*	CBS:125.72	Ukraine	OQ910798	OQ982820	OQ911157	OQ927864
*C. rosea f. catenulata*	CBS:511.66	Russia	OQ910804	OQ982825	OQ911163	OQ927870
*C. rosea f. catenulata*	CBS:569.69	Switzerland	OQ910805	OQ982826	OQ911164	OQ927871
*C. rosea f. catenulata*	CBS 154.27	USA	OQ910800	AF358160	MH866405	OQ927866
*C. agarwalii*	CBS 533.81	India	OQ910526	OQ982571	OQ910885	OQ927604
*C. apocyni*	CBS:956.73	Mexico	OQ910530	OQ982575	OQ910889	OQ927608
*C. apocyni*	CBS:360.78	USA	OQ910529	OQ982574	OQ910888	OQ927607
*C. aureofulvella*	CBS:235.80	New Zealand	OQ910539	OQ982583	OQ910898	OQ927617
*C. aureofulvella*	CBS:102839	Australia	OQ910536	OQ982580	OQ910895	OQ927614
*C. australiana*	CBS 102423	Australia	OQ910541	OQ982585	OQ910900	OQ927619
*C. australiana*	CBS 102421	Australia	OQ910540	OQ982584	OQ910899	OQ927618
*C. bambusae*	CBS 139411	Thailand	OQ910542	OQ982586	OQ910901	OQ927620
*C. capitata*	CBS:218.93	Japan	OQ910545	OQ982589	MH874054	OQ927623
*C. chlorina*	CBS 287.90	Brazil	OQ910546	OQ982590	OQ910905	OQ927624
*C. chloroleuca*	CBS 141592	Brazil	OQ910553	OQ982596	OQ910912	OQ927631
*C. chloroleuca*	CBS:141590	Brazil	OQ910551	OQ982594	OQ910910	OQ927629
*C. compactiuscula*	CBS:123786	USA	OQ910565	OQ982605	OQ910924	OQ927642
*C. compactiuscula*	CBS:123781	USA	OQ910564	OQ982604	OQ910923	OQ927641
*C. compactiuscula*	CBS:123759	USA	OQ910563	OQ982603	OQ910922	OQ927640
*C. divergens*	CBS:532.69	Canada	OQ910574	OQ982611	OQ910933	OQ927650
*C. divergens*	CBS:120975	The Netherlands	OQ910571	OQ982609	OQ910930	OQ927648
*C. ellipsoidea*	CBS 102566	India	OQ910579	OQ982616	OQ910938	OQ927654
*C. ellipsoidea*	CBS:175.76	Indonesia	OQ910580	OQ982617	OQ910939	OQ927655
*C. epichloe*	CBS 101037	Japan	OQ910581	OQ982618	OQ910940	OQ927656
*C. eriocamporesii*	CBS 647.91	Germany	OQ910582	OQ982619	OQ910941	–
*C. eriocamporesii*	MFLUCC 19-0486	Thailand	MN699133	–	MN699128	–
*C. farinosa*	CBS:124067	China	OQ910593	OQ982629	OQ910952	OQ927665
*C. farinosa*	CBS:137553	France	OQ910599	OQ982634	OQ910958	OQ927671
*C. fujianensis*	CBS 127474	China	OQ910620	OQ982655	OQ910979	OQ927691
*C. fusca*	CBS 207.93	French Guiana	OQ910622	OQ982657	OQ910981	OQ927693
*C. fusca*	CBS:101925	USA	OQ910621	OQ982656	OQ910980	OQ927692
*C. grammicospora*	CBS 209.93	French Guiana	OQ910625	OQ982659	OQ910984	OQ927696
*C. grammicosporopsis*	CBS:102834	Australia	OQ910626	OQ982660	OQ910985	OQ927697
*C. grammicosporopsis*	CBS:102843	Australia	OQ910628	OQ982662	OQ910987	OQ927699
*C. grammicosporopsis*	CBS:102835	Australia	OQ910627	OQ982661	OQ910986	OQ927698
*C. hongkongensis*	CBS 115291	China	OQ910630	OQ982663	OQ910989	OQ927700
*C. hongkongensis*	CBS 116542	China	OQ910631	–	OQ910990	OQ927701
*C. intermedia*	CBS 508.82	The Netherlands	OQ910632	OQ982664	OQ910991	–
*C. kowhai*	CBS 461.95	New Zealand	OQ910633	OQ982665	OQ910992	OQ927702
*C. krabiensis*	CBS 192.96	Papua New Guinea	OQ910634	OQ982666	OQ910993	OQ927703
*C. kunmingensis*	YFCC 898	China	MW199069	MW201676	MW199058	–
*C. kunmingensis*	YFCC 892	China	MW199070	MW201677	MW199059	–
*C. kunmingensis*	CBS 101920	Jamaica	OQ910635	OQ982667	OQ910994	OQ927704
*C. levigata*	CBS 101916	France	OQ910636	OQ982668	OQ910995	OQ927705
*C. levigata*	CBS 203.69	UK	OQ910638	OQ982670	OQ910997	OQ927707
*C. lucifer*	CBS:126.87	French Guiana	OQ910645	OQ982676	OQ911004	OQ927714
*C. lucifer*	CBS:100008	USA	OQ910644	OQ982675	OQ911003	OQ927713
*C. miodochialis*	CBS 997.69	The Netherlands	OQ910646	OQ982677	OQ911005	OQ927715
*C. moreaui*	CBS 127881	Spain	OQ910647	OQ982678	OQ911006	OQ927716
*C. oblongispora*	CBS 100285	Japan	OQ910648	OQ982679	OQ911007	OQ927717
*C. obovatispora*	CBS 118752	Germany	OQ910649	OQ982680	OQ911008	OQ927718
*C. palmae*	CBS 119.87	Indonesia	OQ910650	OQ982681	OQ911009	OQ927719
*C. penicillata*	CBS 729.87	Germany	OQ910654	OQ982685	OQ911013	OQ927722
*C. penicillata*	CBS:148211	The Netherlands	OQ910652	OQ982683	OQ911011	OQ927721
*C. pseudochroleuca*	CBS 447.96	USA	OQ910671	OQ982702	OQ911030	OQ927739
*C. pseudochroleuca*	CBS 186.94	The Netherlands	OQ910664	OQ982695	OQ911023	OQ927732
*C. pseudochroleuca*	CBS 124610	Cameroon	OQ910661	OQ982692	OQ911020	OQ927729
*C. pseudostriata*	CBS:120.87	Indonesia	OQ910672	OQ982703	OQ911031	OQ927740
*C. pseudostriata*	CBS:309.96	Papua New Guinea	OQ910673	OQ982704	OQ911032	OQ927741
*C. ralfsii*	CBS:127880	Portugal	OQ910679	OQ982709	OQ911038	OQ927747
*C. reniformis*	CBS 695.86	Unknown	OQ910685	OQ982714	OQ911044	OQ927753
*C. rhizophaga*	CBS 124508	Syria	OQ910688	OQ982717	OQ911047	OQ927756
*C. rhizophaga*	CBS 125416	Italy	OQ910692	OQ982721	OQ911051	OQ927760
*C. rhizophaga*	CBS 124507	Syria	OQ910687	OQ982716	OQ911046	OQ927755
*C. rogersoniana*	CBS:920.9	Brazil	OQ910711	OQ982740	OQ911070	OQ927779
*C. rogersoniana*	CBS:668.70	India	OQ910710	OQ982739	OQ911069	OQ927778
*C. rogersoniana*	CBS:582.89	Brazil	OQ910709	OQ982738	OQ911068	OQ927777
*C. samuelsii*	CBS:701.97	USA	OQ910814	OQ982834	OQ911173	OQ927879
*C. samuelsii*	CBS:199.93	Guyana	OQ910810	OQ982830	OQ911169	OQ927876
*C. solani*	CBS 102418	The Netherlands	OQ910819	OQ982838	OQ911178	OQ927884
*C. sporodochialis*	CBS:133758	French Guiana	OQ910863	OQ982880	OQ911222	OQ927926
*C. sporodochialis*	CBS:101921	USA	OQ910862	OQ982879	OQ911221	OQ927925
*C. subquaternata*	CBS 100003	French Guiana	OQ910865	OQ982882	OQ911224	OQ927928
*C. subquaternata*	CBS:108.87	Venezuela	OQ910867	OQ982883	OQ911226	OQ927930
*C. zelandiaenovae*	CBS 102422	Australia	OQ910873	OQ982887	OQ911232	OQ927936
*C. zelandiaenovae*	CBS 102844	New Zealand	OQ910874	OQ982888	OQ911233	OQ927937
*Stephanonectria chromolaenae*	CBS:476.91	Turkey	OQ911304	OQ968131	OQ911365	OQ914856
*S. chromolaenae*	CBS:475.91	Turkey	OQ911303	OQ968130	OQ911364	OQ914855

– Failed to obtain sequence.

**Table 2:** Plant pathogenic diseases induced by *Clonostachys rosea*.

Host	Country	Disease Symptoms	References
*Hordeum vulgare*	China	Stunted plants, yellow–brown stem base patches, blackened or rotting roots	Li et al. (2022) [36]
*Vicia faba*	Iran	Rotting roots, brown discoloration, soft texture, plant death	Afshari and Hemmati (2017) [37]
*Beta vulgaris*	Morocco	Yellowing and chlorosis of leaves, black to brown necrotic root lesions and rot	Farhaoui et al. (2023) [38]
*Persea americana*	Mexico	Small fruit, with severe cases showing pulp oxidation	Coyotl-Pérez et al. (2022) [39]
*Xanthoceras sorbifolium*	China	Leaves wilt and drop, roots turn brown and necrotic	Li et al. (2024) [40]
*Allium sativum*	Mexico	Stunted growth, plant wilting, smaller bulbs, leaf desiccation, light-brown root lesions and rot	Diaz et al. (2022) [41]
*Angelica sinensis*	China	Root rot with water-soaked lesions, later turning black; vascular browning in stems; plant wilting, yellowing, and growth cessation	Ma et al. (2022) [42]
*Camellia sinensis*	China	Leaf shrinkage and yellowing, circular or irregular brown spots, faded yellow tissue around lesions	Ling et al. (2023) [43]
*Fritillaria taipaiensis*	China	Brown to black spots on roots and bulbs, necrotic rot	Wang et al. (2024) [44]
*Astragalus membranaceus*	China	Yellowing leaves and defoliation, multiple brown longitudinal root cracks or fissures, soft roots, rot, and plant death	Qi et al. (2022) [45]
*Glycine max*	United States	Main and lateral root necrosis	Bienapfl et al. (2012) [46]

## Data Availability

All the relevant data used in this study are included in this manuscript. The corresponding author can be contacted if any further information is needed.
